# Breast Cancer Incidence After a False-Positive Mammography Result

**DOI:** 10.1001/jamaoncol.2023.4519

**Published:** 2023-11-02

**Authors:** Xinhe Mao, Wei He, Keith Humphreys, Mikael Eriksson, Natalie Holowko, Haomin Yang, José Tapia, Per Hall, Kamila Czene

**Affiliations:** 1 Department of Medical Epidemiology and Biostatistics, Karolinska Institutet, Stockholm, Sweden; 2 Chronic Disease Research Institute, the Children’s Hospital, and National Clinical Research Center for Child Health, School of Public Health, School of Medicine, Zhejiang University, Hangzhou, Zhejiang, China; 3 Department of Nutrition and Food Hygiene, School of Public Health, Zhejiang University, Hangzhou, Zhejiang, China; 4 Department of Medicine Solna, Clinical Epidemiology Division, Karolinska Institutet, Stockholm, Sweden; 5 Department of Epidemiology and Health Statistics, School of Public Health, Fujian Medical University, Fuzhou, China; 6 Department of Oncology, Södersjukhuset, Stockholm, Sweden

## Abstract

**Importance:**

False-positive mammography results are common. However, long-term outcomes after a false-positive result remain unclear.

**Objectives:**

To examine long-term outcomes after a false-positive mammography result and to investigate whether the association of a false-positive mammography result with cancer differs by baseline characteristics, tumor characteristics, and time since the false-positive result.

**Design, Setting, and Participants:**

This population-based, matched cohort study was conducted in Sweden from January 1, 1991, to March 31, 2020. It included 45 213 women who received a first false-positive mammography result between 1991 and 2017 and 452 130 controls matched on age, calendar year of mammography, and screening history (no previous false-positive result). The study also included 1113 women with a false-positive result and 11 130 matched controls with information on mammographic breast density from the Karolinska Mammography Project for Risk Prediction of Breast Cancer study. Statistical analysis was performed from April 2022 to February 2023.

**Exposure:**

A false-positive mammography result.

**Main Outcomes and Measures:**

Breast cancer incidence and mortality.

**Results:**

The study cohort included 497 343 women (median age, 52 years [IQR, 42-59 years]). The 20-year cumulative incidence of breast cancer was 11.3% (95% CI, 10.7%-11.9%) among women with a false-positive result vs 7.3% (95% CI, 7.2%-7.5%) among those without, with an adjusted hazard ratio (HR) of 1.61 (95% CI, 1.54-1.68). The corresponding HRs were higher among women aged 60 to 75 years at the examination (HR, 2.02; 95% CI, 1.80-2.26) and those with lower mammographic breast density (HR, 4.65; 95% CI, 2.61-8.29). In addition, breast cancer risk was higher for women who underwent a biopsy at the recall (HR, 1.77; 95% CI, 1.63-1.92) than for those without a biopsy (HR, 1.51; 95% CI, 1.43-1.60). Cancers after a false-positive result were more likely to be detected on the ipsilateral side of the false-positive result (HR, 1.92; 95% CI, 1.81-2.04) and were more common during the first 4 years of follow-up (HR, 2.57; 95% CI, 2.33-2.85 during the first 2 years; HR, 1.93; 95% CI, 1.76-2.12 at >2 to 4 years). No statistical difference was found for different tumor characteristics (except for larger tumor size). Furthermore, associated with the increased risk of breast cancer, women with a false-positive result had an 84% higher rate of breast cancer death than those without (HR, 1.84; 95% CI, 1.57-2.15).

**Conclusions and Relevance:**

This study suggests that the risk of developing breast cancer after a false-positive mammography result differs by individual characteristics and follow-up. These findings can be used to develop individualized risk-based breast cancer screening after a false-positive result.

## Key Points

**Question:**

What are the long-term outcomes for women who receive a false-positive mammography result?

**Findings:**

In this population-based, matched cohort study in Sweden, women with a false-positive mammography result had an elevated incidence of breast cancer and mortality for up to 20 years; the increased breast cancer risk varied according to age, mammographic breast density, and whether a biopsy was performed during recall or not. In addition, the risk of breast cancer was more pronounced during the initial years after the false-positive mammography result.

**Meaning:**

This study suggests that breast cancer awareness should be emphasized long term for women with a false-positive mammography result; developing personalized surveillance programs can be beneficial for these women.

## Teaser

This Swedish cohort study examines long-term outcomes after a false-positive mammography result and investigates whether the association of a false-positive mammography result with cancer differs by baseline characteristics, tumor characteristics, and time since the false-positive result.

## Introduction

Mammography screening programs are associated with a reduction in breast cancer mortality of more than 20%.^1,2,3^ One of the harms of mammography screening is the occurrence of a false-positive mammography result.^4,5,6^ In the US, approximately 11% of women receive a false-positive result from a single screening,^7^ while in Europe, the corresponding proportion is approximately 2.5%.^6,8^ Despite the lower rate in Europe, this still corresponds to a large cumulative risk of having a false-positive result; after 10 screenings, approximately 1 in 5 women in Europe will experience at least 1 false-positive mammography result.^6,9,10^ Given that false-positive results may lead to psychological distress and anxiety, they may also influence attendance rates and jeopardize the success of screening programs.^5,7,11,12^ Therefore, false-positive results represent a critical public health issue.

Apart from psychological stress, previous studies consistently show that women with false-positive results have an increased risk of developing breast cancer within 10 years of follow-up compared with women without false-positive results.^12,13,14,15,16^ However, to our knowledge, little is known about long-term outcomes after a false-positive mammography result and whether the risk of breast cancer differs by individual characteristics. For example, given that breast density is associated with mammography screening performance,^17^ women with high and low breast density may be recalled due to different reasons and thus have different risks of subsequent breast cancer. In addition, although a false-positive result has been shown to be associated with an elevated risk for both mammography-detected and interval cancers to the same degree,^16,18^ it is unclear whether the risk associated with breast cancer may differ for different breast cancer subtypes.

Using data from the Stockholm Mammography Screening program and linkages to Swedish nationwide registers, we investigated long-term outcomes after a false-positive mammography result. Specifically, this study investigated whether there is a long-term increased risk of breast cancer among women with a false-positive mammography result and whether this risk differs by individual characteristics. In addition, we also investigated the association between a false-positive mammography result and mortality.

## Methods

### Data Source and Study Population

The Stockholm Mammography Screening program started in 1989, inviting all women aged 50 to 69 years to be screened every 2 years. From 2005 to 2012, women aged 40 to 49 years were invited to be screened at an interval of 18 months. From 2012 onward, this was changed to a 2-year interval; additionally, the program began inviting women aged 70 to 74 years for screening. For each woman and at each screening, mammograms were independently read by 2 radiologists to decide whether to recall the woman for further examinations.^8^ Whenever there were diverging opinions, the radiologists discussed until a consensus was reached. Typically, women who were recalled received a letter within 1 week.^8^ Our data set shows that 99.3% of these women attended the follow-up evaluation. Furthermore, among those who returned for the evaluation, 50.0% did so within 13 days and 90.0% within 27 days from their initial mammography. This cohort study was approved by the regional ethical board at Karolinska Institutet, Stockholm, Sweden. Written informed consent was obtained from all Karolinska Mammography Project for Risk Prediction of Breast Cancer (KARMA) study participants included in this study. The study adhered to the Strengthening the Reporting of Observational Studies in Epidemiology (STROBE) reporting guideline for cohort studies.^19^

Using the unique Swedish Personal Identification Number system, we linked the Stockholm Mammography Screening Register to other Swedish registers. Our population was drawn from 596 270 women aged 40 to 74 years who had ever undergone a mammography screening in the Stockholm area between 1991 and 2017. After excluding women who received a diagnosis of breast cancer before 1991 (n = 2384), our final study population included 593 886 women and a total of 2 635 668 screening records (eFigure in Supplement 1). On average, each woman attended 4 screenings throughout the study period. We identified 45 213 women without breast cancer who received a first false-positive result. Using a matched cohort design,^20^ for each woman, we randomly selected 10 controls who were not recalled at that screening, matching (with replacement)^21^ on age, calendar year of mammography, and screening history (no previous false-positive result). The side (left or right) that was recalled for each woman with a false-positive result was correspondingly assigned as the “recalled side” for the matched controls.

### Exposure

Having a false-positive result at a mammography screening was defined as having a mammography result with a positive interpretation but without a breast cancer diagnosed before the next scheduled screening or end of a normal screening interval. These data were sourced from the Stockholm Mammography Screening program.^8^

### Outcomes

Information on date of cancer diagnosis, side of breast cancer, type of breast cancer (invasive or in situ), tumor size (<20 or ≥20 mm), lymph node involvement (no or yes), estrogen receptor status (positive or negative), progesterone receptor status (positive or negative), ERBB2 status (positive or negative), tumor grade (I, II, or III), and Ki-67 status was retrieved from the Stockholm-Gotland Breast Cancer Register. Molecular subtypes of breast cancer—luminal-A, luminal-B, and triple-negative breast cancer—were defined based on estrogen receptor, progesterone receptor, ERBB2, tumor grade, and Ki-67 status.^22^ We defined screening-detected cancers as breast cancers detected after a positive screening result and before the next scheduled screening or end of a normal screening interval. We defined interval cancers as breast cancers detected after a negative mammography result at screening and before the next scheduled screening or end of a normal screening interval. Date and cause of death were determined through the Swedish Cause of Death Register.

To investigate breast cancer incidence after a false-positive result, we followed up with women from the date of the next screening after the index mammography until the first diagnosis of breast cancer, death, emigration, or March 31, 2020, whichever came first. Similarly, to investigate mortality after a false-positive result, we followed up with women until death, emigration, or March 31, 2020, whichever came first.

### Covariates

A positive family history of breast cancer was determined based on having mothers and sisters (identified through the Multi-Generation Register) who received a diagnosis of breast cancer (extracted from the Swedish Cancer Register) before the time of the index mammography. Educational level (≤9, 10-12, or >12 years) was defined using the highest level of education recorded in the LISA (Longitudinal Integrated Database for Health Insurance and Labour Market Studies) database. Women with a false-positive result were stratified by whether they had a cytologic aspiration biopsy or not when recalled for further examinations. Of the 45 213 women with a false-positive result, 11 463 (25.4%) underwent a biopsy at recall. Information on mammographic breast density was available from the KARMA study.^23^ Percentage mammographic breast density of women in the KARMA study at each screening was measured using the STRATUS method^24^ and categorized into BI-RADS (Breast Imaging Reporting and Data System) scores.^25^ Hereafter, we refer to the computer-generated score as the cBI-RADS score.

### Statistical Analysis

Statistical analysis was performed from April 2022 to February 2023. We used the Nelson-Aalen estimator to examine the cumulative incidence of breast cancer for women with a false-positive result and for the controls (screened women who were not recalled). We used Cox proportional hazards regression analysis to investigate whether the breast cancer risk differed by baseline characteristics (interaction analyses) or subtypes of breast cancer. Cox proportional hazards regression models were stratified by matching identifiers in all analyses, which allows for the interpretation of our results independently of the matching variables (specifically, age and calendar year). We additionally adjusted for family history of breast cancer and educational level in our analyses. We hypothesized that the association between false-positive results and the subsequent risk of breast cancer differs by breast side and duration of follow-up. Thus, we fitted flexible parametric models^26,27^ to investigate the risk of breast cancer after a false-positive result by the side on which the breast cancer was detected (ipsilateral or contralateral to the false-positive result). Finally, we used Cox proportional hazards regression models to examine the association between having a false-positive result and breast cancer–specific and overall mortality.

In this study, all *P* values were 2-sided, and results were considered statistically significant at *P* < .05. The analyses were conducted in SAS, version 9.4 (SAS Institute Inc); Stata, version 17.0 (StataCorp LLC); and R, version 4.0.5 (R Group for Statistical Computing). Using data from the high-quality Swedish registers and KARMA cohort, we achieved virtually complete follow-up, with most variables featuring less than 5% missing values.^8,23,28,29,30^

## Results

**Figure 1.**
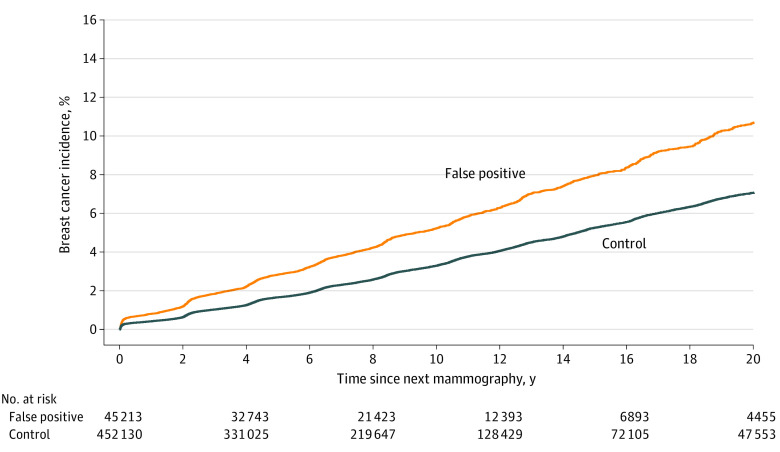
Cumulative Breast Cancer Incidence Among Women With vs Without a False-Positive Result at Index Mammography Women were followed up from the date of their next screening after their index mammography.

The study cohort included 497 343 women (median age, 52 years [IQR, 42-59 years]). At the index mammography, most women included in this study were older than 50 years (321 112 [64.6%]), born in Sweden (383 203 [77.1%]), without a family history of breast cancer (353 553 of 383 203 [92.3%]) (eTable 1 in Supplement 1). The 20-year cumulative incidence of breast cancer was 11.3% (95% CI, 10.7%-11.9%) among women with a false-positive result. The corresponding cumulative incidence among women without a false-positive result was 7.3% (95% CI, 7.2%-7.5%) (Figure 1). The association between breast cancer risk and having a false-positive result was still apparent after adjusting for covariates; we estimated an adjusted hazard ratio (HR) of 1.61 (95% CI, 1.54-1.68) for women with a false-positive result compared with those without.

In interaction analyses, we found that the association between false-positive results and breast cancer risk was statistically significantly higher among women aged 60 to 75 years (HR, 2.02; 95% CI, 1.80-2.26) compared with women aged 40 to 49 years (HR, 1.38; 95% CI, 1.23-1.56) (Table 1).^23^ This association did not differ significantly by calendar year, family history of breast cancer, or educational level.^23^ We additionally found that the risk was statistically significantly higher among women with lower breast density (cBI-RADS A and B) (HR, 4.65; 95% CI, 2.61-8.29) compared with women with higher breast density (cBI-RADS C and D) (HR, 1.60; 95% CI, 0.93-2.73). When stratified based on whether a biopsy was performed during the recall, we observed that women who underwent a biopsy had a higher risk of breast cancer (HR, 1.77; 95% CI, 1.63-1.92) than those without a biopsy (HR, 1.51; 95% CI, 1.43-1.60) when compared with women who were not recalled.

**Table 1.** Risk of Breast Cancer After a False-Positive Mammography Result, by Baseline Characteristics

Characteristic	Women, No.	Breast cancer cases diagnosed during follow-up, No.	Hazard ratio (95% CI)^a^	*P* value for interaction^b^
Age at index mammography, y^c^				
40-49	176 231	2486	1.38 (1.23-1.56)	<.001
50-59	103 840	2419	1.53 (1.36-1.72)	
60-75	74 250	2156	2.02 (1.80-2.26)	
Calendar year at index mammography^d^				
1991-1999	95 755	3424	1.70 (1.54-1.87)	.45
2000-2008	98 923	3732	1.63 (1.48-1.79)	
2009-2017	113 080	2110	1.80 (1.59-2.03)	
Family history of breast cancer				
Without	353 553	12 357	1.58 (1.50-1.67)	.66
With	29 650	1299	1.51 (1.27-1.81)	
Educational level, y				
≤9	74 101	3007	1.68 (1.50-1.88)	.45
10-12	197 549	6846	1.54 (1.43-1.66)	
>12	223 340	6890	1.59 (1.47-1.71)	
Mammographic density at index mammography^e^				
Low (cBI-RADS A and B)	5108	67	4.65 (2.61-8.29)	.01
High (cBI-RADS C and D)	7135	108	1.60 (0.93-2.73)	

Abbreviations: cBI-RADS, computer-generated Breast Imaging Reporting and Data System density score; KARMA, Karolinska Mammography Project for Risk Prediction of Breast Cancer.

^a^ Estimated by stratified Cox proportional hazards regression models.

^b^ Estimated by adding an interaction term in the stratified Cox proportional hazards regression models.

^c^ For analyses of age, only a mammography performed in 2005 onward was included. Because women aged 40 to 49 years were invited to screening from 2005 onward, by doing this, we made the subgroup analyses comparable.

^d^ For analyses of calendar year, only mammography results from women aged 50 to 69 years were included, with a follow-up no longer than 10 years. Only women aged 50 to 69 years were invited to screening in all studied calendar years, and women who underwent mammography at an earlier rather than later calendar year had a longer follow-up time. By having these restrictions, we made the subgroup analyses comparable.

^e^ Analyses were conducted among women in the KARMA study with available information on mammographic breast density. Their density was classified into cBI-RADS scores: A (<2%), B (2%-16.9%), C (17%-48.9%), and D (≥49%). The KARMA study invited women who attended mammography screening or clinical mammography between 2011 and 2013, and 32 185 women from Stockholm agreed to participate.^23^ Specifically, we included 1113 women from the KARMA study who received their first false-positive results between 2011 and 2015 and were aged 40 to 74 years at mammography and 11 130 matched controls. We followed up with these women until first diagnosis of breast cancer, death, emigration, or December 31, 2019, whichever came first.

**Figure 2.**
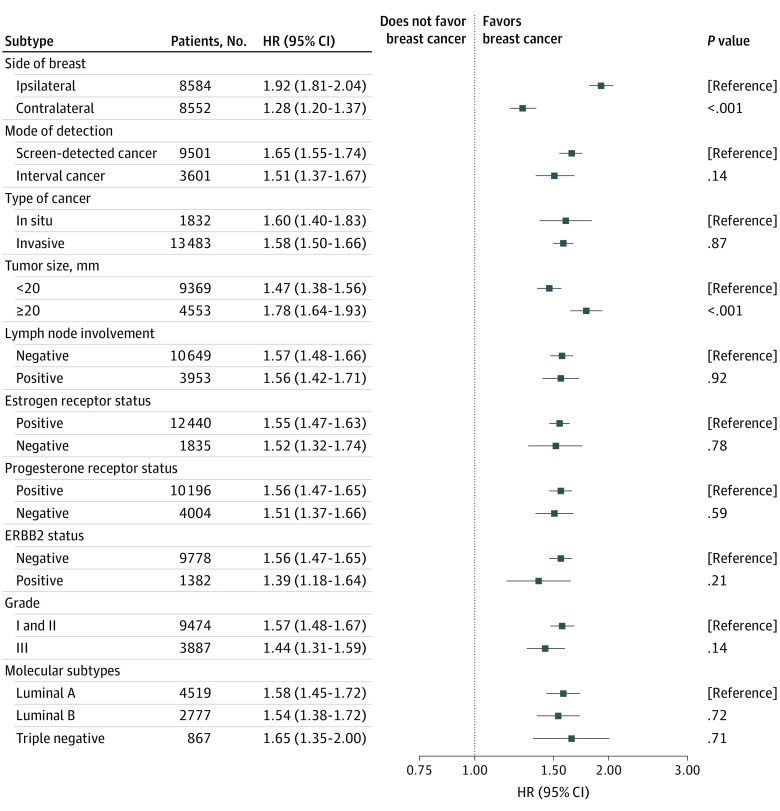
Hazard Ratios (HRs) for Breast Cancer After a False-Positive Result, by Tumor Characteristics HRs and 95% CIs are estimated by stratified Cox proportional hazards regression models. *P* values are derived from the *z* score for differences in HRs from baseline categories. Luminal A subtype was defined as ERBB2 negative, estrogen receptor (ER) positive, grade I, or ERBB2 negative, ER positive, grade II, Ki-67 ≤10%, or ERBB2 negative, ER negative, grade II, 11% ≤ Ki-67 ≤ 20%, progesterone receptor (PR) ≥20%; luminal B subtype was defined as ERBB2 negative, ER positive, grade II, 11% ≤ Ki-67 ≤ 20%, PR <20%, or ERBB2 negative, ER positive, grade II, Ki-67 ≥21%, or ERBB2 negative, ER positive, grade III; triple-negative subtype was defined as ERBB2 negative, ER negative, and PR negative.

We further found that the association between having a false-positive result and breast cancer incidence was similar between breast cancer subtypes—with the exception of the laterality of breast cancer and tumor size (Figure 2). The adjusted HR for having a breast cancer ipsilateral to the false-positive result was 1.92 (95% CI, 1.81-2.04) compared with women with and women without a false-positive result. This was statistically significantly higher than the estimated HR of 1.28 (95% CI, 1.20-1.37) for having a breast cancer on the contralateral side. Compared with women without a false-positive result, women with a false-positive result were more likely to have a larger tumor (≥20 mm); the estimated adjusted HR was 1.78 (95% CI, 1.64-1.93), which was higher than the HR of 1.47 (95% CI, 1.38-1.56) for having a smaller tumor (<20 mm). Our data showed that the screening attendance rate at the next screening was statistically lower for women with a false-positive result (84.6%; 95% CI, 84.3%-84.9%) compared with those without a false-positive result (86.5%; 95% CI, 86.4%-86.6%).

**Figure 3.**
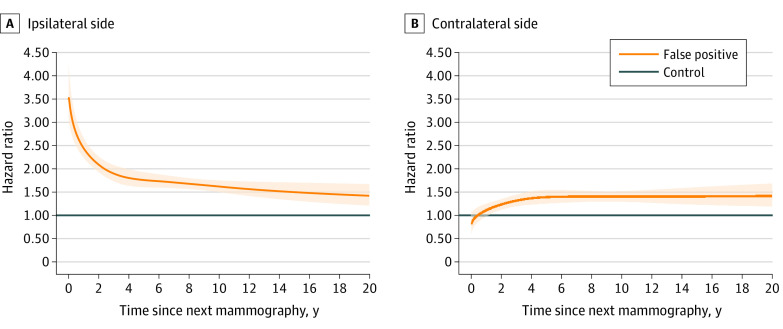
Hazard Ratios for Breast Cancer After a False-Positive Result, by Side and by Follow-Up Time Hazard ratios and 95% CIs are estimated by flexible parametric models, adjusting for age and calendar year of mammography, family history of breast cancer, and educational level. The shaded area indicates 95% CIs.

Furthermore, we examined whether there were time-dependent features of the distinct breast cancer risks on the ipsilateral and contralateral sides to the false-positive result. Compared with women without a false-positive result, the HRs of breast cancer detected in women on the ipsilateral side to the false-positive result decreased sharply in the first several years of follow-up and decreased gradually afterward: 2.57 (95% CI, 2.33-2.85) during the first 2 years, 1.93 (95% CI, 1.76-2.12) at 2 to 4 years, 1.69 (95% CI, 1.57-1.82) at 6 to 10 years, and 1.51 (95% CI, 1.33-1.72) at 10 to 20 years of follow-up (Figure 3; eTable 2 in Supplement 1). In contrast, there was no apparent increase in the risk of a breast cancer being detected on the contralateral side within the first 2 years of follow-up (HR, 1.08; 95% CI, 0.94-1.23); this risk did, however, increase after the initial subsequent years and showed a stable HR of approximately 1.4 up to 20 years of follow-up (4-6 years: HR, 1.38; 95% CI, 1.25-1.54; 6-10 years: HR, 1.40; 95% CI, 1.28-1.53; 10-20 years: HR, 1.40; 95% CI, 1.23-1.60). Women with a false-positive result were at increased risk of all-cause mortality and death due to breast cancer, with HRs of 1.07 (95% CI, 1.04-1.11) and 1.84 (95% CI, 1.57-2.15), respectively (Table 2). Comparing the prognosis of patients with breast cancer with and without a false-positive result gave an HR of 1.05 (95% CI, 0.89-1.25).

**Table 2.** Risk of Death After a False-Positive Result

Outcome	Women, No.	Deaths during follow-up, No.	Hazard ratio (95% CI)^a^
All-cause mortality			
Controls	452 130	38 823	1.00 [Reference]
Women with a false-positive result	45 213	4064	1.07 (1.04-1.11)
Death due to breast cancer			
Controls	452 130	1055	1.00 [Reference]
Women with a false-positive result	45 213	191	1.84 (1.57-2.15)

^a^ Estimated by stratified Cox proportional hazards regression models, additionally adjusting for family history of breast cancer and educational level.

## Discussion

In this large population-based cohort study with a long follow-up, we found that women with a false-positive mammography result had an increased risk of subsequent breast cancer, persisting up to 20 years. The increased risk differed by host characteristics; we observed a higher risk among women aged 60 to 75 years, women who underwent a biopsy at recall, and women with low mammographic breast density. In addition, we found higher risk for large tumors (≥20 mm) and tumors on the ipsilateral side to the false-positive result. An increased risk for breast cancer on the ipsilateral side was highest within the first 4 years of follow-up, while a stable long-term increased risk was also observed for cancers on the contralateral side. In addition, we observed that women with a false-positive result were at increased risk of death from breast cancer.

Our results are in line with previous findings^31,32^ that the increased breast cancer risk associated with having a false-positive result was lower for women without a biopsy. In addition, when comparing women with and women without a false-positive result, we found that the increased risk of breast cancer was less pronounced among young women (aged 40-49 years) and among women with higher mammographic breast density. Because high mammographic breast density may mask tumors and make mammography results difficult to assess,^17^ we hypothesize that women with dense breasts are more likely to be recalled due to a masking effect. In contrast, results for women with nondense breasts are more likely to be a consequence of suspicious findings and are thus associated with a higher increased risk of breast cancer compared with results for women with dense breasts.

In line with previous studies,^12,33^ our results show that women with a false-positive mammography result are more likely to have larger rather than smaller tumors at diagnosis compared with women without a false-positive result. High mammographic breast density may partly explain this finding, given that previous research has established an association between dense breasts and a false-positive result, as well as between dense breasts and larger tumor size.^5,34,35^ In addition, larger tumors might result from a reluctance to undergo screening after a false-positive result, as we found that the next screening’s attendance rate was lower for women with a false-positive result compared with those without one.

We did not find any association between a false-positive mammography result and other tumor characteristics or molecular subtypes. Given the narrow 95% CIs of the HRs, our findings suggest that false-positive results are not associated with tumor characteristics of subsequent cancers, aside from tumor size.

Two mechanisms can potentially explain the increased risk of breast cancer after false-positive results. The first is that the increased risk could be due to small tumors being missed at the previous mammography^36,37^ or to proliferative benign breast disease among women with a false-positive result.^16,37^ Our results, which show an elevated risk of breast cancer on the ipsilateral side that attenuated over time and was highest within the first 4 years of follow-up, support this hypothesis. These results highlight the importance of conducting further investigations into whether a short-term and intensive surveillance program might benefit women with a false-positive result.

Alternatively, a false-positive result may serve as an indicator of a generally higher risk of breast cancer. After the first 4 years of follow-up, we observed a similar and long-term increased risk of breast cancer on the ipsilateral and contralateral sides among women with a false-positive result compared with those without. This finding is partly associated with higher mammographic breast density among women with false-positive results.^17^ Further studies are needed to determine other factors explaining this long-term risk, such as hormone-related or genetic factors.

Furthermore, to our knowledge, this is the first study to show that women with a false-positive result are at increased risk of death from breast cancer. This increased breast cancer mortality was most probably associated with the increased breast cancer incidence, as the prognosis of patients with breast cancer did not differ based on whether or not they had false-positive results before. Comparing the prognosis of patients with breast cancer with a false-positive result with the prognosis of patients with breast cancer without a false-positive result gave an HR of 1.05 (95% CI, 0.89-1.25). Given that false-positive results are common (ie, approximately half [49.0%] of women in the US and 20.0% of women in Europe will have at least 1 false-positive result after 10 screenings),^6,38^ our findings emphasize that they are also a critical public health issue.

### Limitations and Strengths

This study has some limitations. The generalizability of our findings to other countries could be limited given that screening intervals, false-positive rates, and strategies vary from country to country. Considering that the factors associated with a false-positive result have been identified as similar in Europe and the US,^5,39^ we believe that the findings of our study may be applicable to the US context. Still, the magnitude of the increased breast cancer risk and mortality rate needs to be evaluated using data from the US, considering the differences in false-positive rates between the US and Europe. Another potential issue should be considered when interpreting our findings. Our results for long-term outcomes after a false-positive result should be primarily interpreted for women aged 50 years or older. Results for younger women should be interpreted with caution because in Stockholm, women aged 40 to 49 years were included in the screening program from 2005 onward.

This study also has some strengths. A major strength of this study is that it is based on the Stockholm Mammography Screening Register, which includes data on all women who have ever undergone a mammography screening in Stockholm. Moreover, with linkages between Swedish registers, we have virtually complete data and a long follow-up. In addition, we have very detailed data on tumor characteristics.

## Conclusions

We note 3 results of our cohort study with clear clinical implications. First, besides having a biopsy, age at a false-positive mammography result and mammographic breast density should be considered in individualizing surveillance programs among women with a false-positive result. Second, close and intensive surveillance within the next 2 screening rounds may be of particular value. Third, long-term awareness of the disease should be promoted among women with a false-positive result to help address the increased risk of breast cancer incidence and mortality among these women. As women age and are eventually no longer invited for screening (in most countries with mammography screening programs, this stops at 70 years of age), it may be worth studying whether women with a false-positive result will benefit from a prolonged screening program.

## Notes

Supplement 1. **eFigure.** Sample Attrition and Study Population Used for Analyses **eTable 1.** Baseline Characteristics of Women With and Without a False-Positive Result **eTable 2.** Risk of Breast Cancer After a False-Positive Result, by Side and by Follow-Up Time

Supplement 2.
Data Sharing Statement
