# Supplementary material for: Breast Cancer Incidence After a False-Positive Mammography Result
Source: JAMA Oncol. 2023 Nov 2;10(1):63–70. doi: 10.1001/jamaoncol.2023.4519 (PMC10623302; doi:10.1001/jamaoncol.2023.4519)
Supplement: Supplement 1. — eFigure. Sample Attrition and Study Population Used for Analyses eTable 1. Baseline Characteristics of Women With and Without a False-Positive Result eTable 2. Risk of Breast Cancer After a False-Positive Result, by Side and by Follow-Up Time [file jamaoncol-e234519-s001.pdf]

Supplementary Online Content

Mao X, He W, Humphreys K, et al. Breast cancer incidence after a false-positive mammography result. *JAMA Oncol.* Published online November 2, 2023. doi:10.1001/jamaoncol.2023.4519

eFigure. Sample Attrition and Study Population Used for Analyses

eTable 1. Baseline Characteristics of Women With and Without a False-Positive Result

eTable 2. Risk of Breast Cancer After a False-Positive Result, by Side and by Follow-Up Time

This supplementary material has been provided by the authors to give readers additional information about their work.

© 2023 Mao X et al. *JAMA Oncology.*

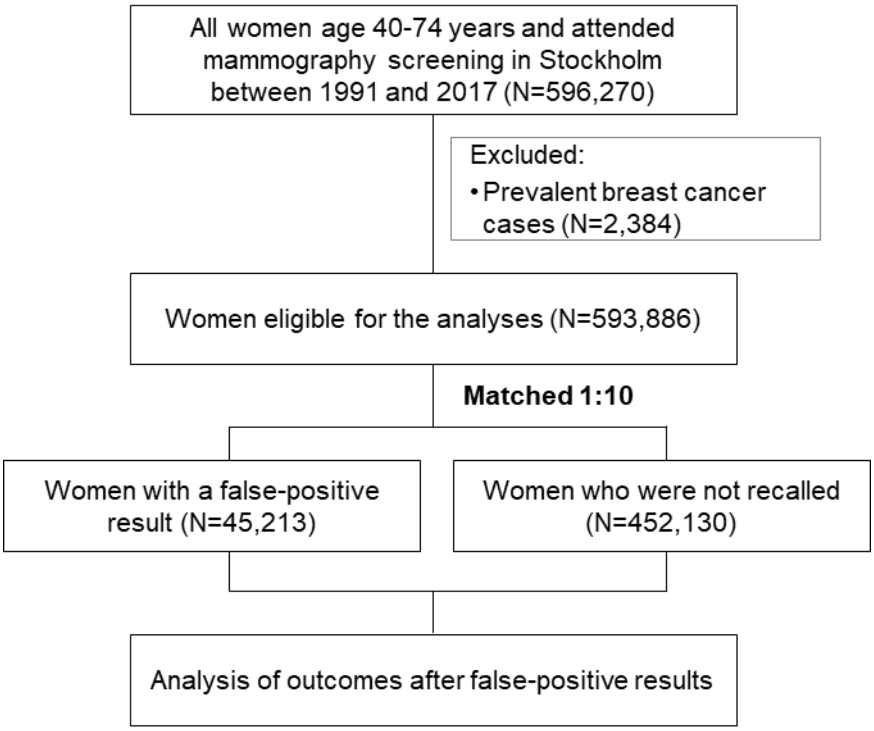

eFigure. Sample Attrition and Study Population Used for Analyses

A false-positive result is defined as a mammogram with a positive interpretation but with no breast cancer diagnosed before the next scheduled screens. Women who were not recalled are defined as those without a result and with no breast cancer diagnosed before the next scheduled screens. All women were followed from the next scheduled screens until outcomes of interest (breast cancer diagnosis or death), emigration, or March 31st, 2020, whichever came first.

© 2023 Mao X et al. *JAMA Oncology.*

eTable 1. Baseline Characteristics of Women With and Without a False-Positive Result

	False-positive results	
	Yes (%)	No (%)
Age at index mammograms (years)		
40-49	16,021 (35.4)	160,210 (35.4)
50-59	18,290 (40.5)	182,900 (40.5)
60-74	10,902 (24.1)	109,020 (24.1)
Calendar year at index mammograms		
1991-1999	8,726 (19.3)	87,260 (19.3)
2000-2008	11,218 (24.8)	112,180 (24.8)
2009-2017	25,269 (55.9)	252,690 (55.9)
Born in Sweden		
No	10,765 (23.8)	103,375 (22.9)
Yes	34,448 (76.2)	348,755 (77.1)
Education (years)		
≤9	6,599 (14.7)	67,502 (15.0)
10-12	17,995 (40.0)	179,554 (39.9)
>12	20,351 (45.3)	202,989 (45.1)
Family history of breast cancer [a]		
No	31,551 (91.6)	322,002 (92.3)
Yes	2,897 (8.4)	26,753 (7.7)

Note: [a] Among women who were born in Sweden.

© 2023 Mao X et al. *JAMA Oncology.*

eTable 2. Risk of Breast Cancer After a False-Positive Result, by Side and by Follow-Up Time

Time since next mammograms (years)	Ipsilateral		Contralateral	
	No. of breast cancer cases diagnosed during follow-up	HR (95%CI) [a]	No. of breast cancer cases diagnosed during follow-up	HR (95%CI) [a]
**(0-2]**	1,697	**2.57 (2.33-2.85)**	1,627	1.08 (0.94-1.23)
**(2-4]**	1,363	**1.93 (1.76-2.12)**	1,352	**1.30 (1.18-1.44)**
**(4-6]**	1,175	**1.77 (1.62-1.95)**	1,199	**1.38 (1.25-1.54)**
**(6-10]**	1,863	**1.69 (1.57-1.82)**	1,849	**1.40 (1.28-1.53)**
**(10-20]**	2,195	**1.51 (1.33-1.72)**	2,204	**1.40 (1.23-1.60)**

Note: Since screening affects the timing of breast cancer diagnosis, instead of calculating the hazards ratio at specific time points, we used flexible parametric models to calculate the average hazards ratios for the different periods during follow-up (0-2, 2-4, 4-6, 6-10, and 10-20 years).

[a] Average hazard ratios (95% confidence interval) for different time intervals are estimated in flexible parametric models, adjusting for age and calendar year on mammograms, family history of breast cancer and education level.

© 2023 Mao X et al. *JAMA Oncology.*
