# Supplementary material for: Breast Cancer Incidence After a False-Positive Mammography Result
Source: JAMA Oncol. 2023 Nov 2;10(1):63–70. doi: 10.1001/jamaoncol.2023.4519 (PMC10623302; doi:10.1001/jamaoncol.2023.4519)
Supplement: Supplement 2. — Data Sharing Statement [file jamaoncol-e234519-s002.pdf]

Data Sharing Statement

Mao. Breast Cancer Incidence After a False-Positive Mammography Result. *JAMA Oncol*. Published November 02, 2023. doi:10.1001/jamaoncol.2023.4519

Data
Data available: No

Additional Information
Explanation for why data not available: Access to these data is subject to certain restrictions. The data was sourced from Statistics Sweden (scb.se), the National Board of Health and Welfare (socialstyrelsen.se), the Regional Cancer Registry (cancercentrum.se), and Karma (karmastudy.org). Researchers can fully access the data upon approval of their applications.
